# A segregação residencial socioeconômica está associada aos problemas do sono? *Insights* do ELSA-Brasil

**DOI:** 10.1590/0102-311XPT111323

**Published:** 2024-07-29

**Authors:** Leonardo Shigaki, Letícia de Oliveira Cardoso, Aline Silva-Costa, Sandhi Maria Barreto, Luana Giatti, Maria de Jesus Mendes da Fonseca, Rosane Harter Griep

**Affiliations:** 1 Escola Nacional de Saúde Pública Sergio Arouca, Fundação Oswaldo Cruz, Rio de Janeiro, Brasil.; 2 Universidade Federal do Triângulo Mineiro, Uberaba, Brasil.; 3 Faculdade de Medicina, Universidade Federal de Minas Gerais, Belo Horizonte, Brasil.; 4 Instituto Oswaldo Cruz, Fundação Oswaldo Cruz, Rio de Janeiro, Brasil.

**Keywords:** Duração do Sono, Insônia, Sonolência Diurna, Vizinhança, Segregação Residencial, Sleep Duration, Insomnia, Daytime Somnolence, Neighborhood, Residential Segregation, Duración del Sueño, Insomnio, Somnolencia Diurna, Vecindarios, Segregación Residencial

## Abstract

O sono é influenciado por diversos fatores e é essencial para a saúde. O papel do contexto socioeconômico da vizinhança na saúde do sono foi estudado nos últimos anos, mas os resultados são inconsistentes. O objetivo deste estudo foi investigar a associação entre a segregação residencial socioeconômica e os problemas do sono. Utilizou-se dados da 2ª avaliação (2012-2014) de 9.918 servidores públicos participantes do *Estudo Longitudinal de Saúde do Adulto* (ELSA-Brasil). A segregação residencial socioeconômica foi avaliada por meio da estatística Getis-Ord Local Gi*, e a duração e privação do sono, as queixas de insônia e a sonolência diurna foram obtidas por meio de entrevistas. Para as estimativas da *odds ratio* (OR), foram utilizados modelos de regressão logística binomial e multinomial. Em relação ao sono, 49% tinham curta duração e 3% longa duração, 23% relataram queixas de insônia, 45% relataram privação do sono, 42% relataram sonolência diurna e 48% relataram ≥ 2 problemas do sono. No modelo ajustado por variáveis demográficas e socioeconômicas, houve associação entre alta segregação residencial socioeconômica e duração curta do sono (OR = 1,22; IC95%: 1,07; 1,40), privação do sono (OR = 1,20; IC95%: 1,05; 1,37), sonolência diurna (OR = 1,17; IC95%: 1,03; 1,34) e ≥ 2 problemas associados do sono (OR = 1,24; IC95%: 1,08; 1,41). Indivíduos que vivem em vizinhanças com alta segregação residencial socioeconômica apresentam maior chance de terem curta duração, privação do sono, sonolência diurna e ≥ 2 problemas associados ao sono. Essas informações reforçam que políticas públicas para reduzir as desigualdades socioeconômicas podem contribuir para melhorar a saúde do sono da população.

## Abstract (en)

Several factors influence sleep, which is essential for health. While the role of neighborhood socioeconomic context on sleep health has been studied in recent years, results are inconsistent. The study aimed to investigate the association between socioeconomic residential segregation and sleep problems, using data from the second evaluation (2012-2014) of 9,918 public servants participating in the *Brazilian Longitudinal Study of Adult Health* (ELSA-Brasil). Socioeconomic residential segregation was assessed using the Getis-Ord Local Gi* statistic. Sleep duration and deprivation, complaints of insomnia, and daytime sleepiness were obtained through interviews. Binomial and multinomial logistic regression models were used to estimate the odds ratio (OR). Regarding sleep, 49% had short duration and 3% long duration, 23% reported complaints of insomnia, 45% sleep deprivation, 42% daytime sleepiness, and 48% reported ≥ 2 sleep problems. In the model adjusted for demographic and socioeconomic variables, there was an association between high socioeconomic residential segregation and short sleep duration (OR = 1.22; 95%CI: 1.07; 1.40), sleep deprivation (OR = 1.20; 95%CI: 1.05; 1.37), daytime sleepiness (OR = 1.17; 95%CI: 1.03; 1.34) and ≥ 2 associated sleep problems (OR = 1.24; 95%CI: 1.08; 1.41). Individuals living in neighborhoods with high socioeconomic residential segregation are more likely to have short sleep duration, sleep deprivation, daytime sleepiness, and ≥ 2 associated sleep problems. This information reinforces that public policy measures to reduce socioeconomic inequalities can improve the population’s sleep health.

## Resumen

El sueño se influye por varios factores y es esencial para la salud. Se estudió el papel del contexto socioeconómico del barrio en la salud del sueño en los últimos años, pero los resultados son inconsistentes. El objetivo del estudio fue investigar la asociación entre la segregación residencial socioeconómica y los problemas de sueño. Se utilizó datos de la 2ª evaluación (2012-2014) de 9918 servidores públicos participantes del *Estudio Longitudinal de Salud del Adulto* (ELSA-Brasil). Se evaluó la segregación residencial socioeconómica a través de la estadística Getis-Ord Local Gi*. La duración y privación del sueño, las quejas de insomnio y somnolencia diurna se obtuvieron a través de entrevista. Se utilizaron modelos de regresión logística binomial y multinominal para estimar el *odds ratio* (OR). Con respecto al sueño, el 49% tenía una duración corta y el 3% tenía una duración larga, el 23% relató quejas de insomnio, el 45% relató privación de sueño, el 42% relató somnolencia diurna y el 48% relató ≥ 2 problemas de sueño. En el modelo ajustado por variables demográficas y socioeconómicas, hubo una asociación entre la alta segregación residencial socioeconómica y la duración corta de sueño (OR = 1,22; IC95%: 1,07; 1,40), la privación de sueño (OR = 1,20; IC95%: 1,05; 1,37), la somnolencia diurna (OR = 1,17; IC95%: 1,03; 1,34) y ≥ 2 problemas asociados con el sueño (OR = 1,24; IC95%: 1,08; 1,41). Personas que viven en barrios con una alta segregación residencial socioeconómica presentan una mayor probabilidad de tener duración corta del sueño, privación de sueño, somnolencia diurna y ≥ 2 problemas asociados con el sueño. Estas informaciones resaltan que medidas de políticas públicas para reducir las desigualdades socioeconómicas pueden contribuir a mejorar la salud del sueño en la población.

## Introdução

O sono é essencial para a vida humana, sendo influenciado pelo ambiente e estando sujeito a fatores interpessoais e sociais ^1^. A boa saúde do sono é resultante de um padrão multidimensional complexo de variação do sono-vigília, sendo definida pela satisfação subjetiva, duração adequada, alta eficiência e estado de alerta sustentado durante as horas de vigília ^2^.

Problemas como curta duração e baixa qualidade do sono têm sido associados com diversos desfechos negativos de saúde, tal qual problemas cognitivos, psicossociais e cardiometabólicos, além de aumentar o risco de condições específicas como diabetes mellitus, doenças cardiovasculares e obesidade ^3^.

Nas últimas décadas, a pesquisa sobre os problemas do sono e seus determinantes individuais e contextuais tem crescido, procurando identificar grupos sociais que estão em maior risco e entender como o contexto do ambiente físico e social da vizinhança atuam na saúde do sono. Como os fatores individuais e contextuais, como as vizinhanças e seus relacionamentos, influenciam o sono, identificar grupos sociais que estão em risco de desenvolver problemas nesse âmbito e entender os mecanismos subjacentes pode ser importante para reduzir as disparidades e promover a saúde do sono ^4^. Nesse sentido, as vizinhanças surgiram como contextos potencialmente relevantes, porque têm atributos físicos e sociais que são capazes de afetar plausivelmente a saúde dos indivíduos ^5^ ^,^ ^6^.

O papel do contexto socioeconômico da vizinhança na saúde do sono foi estudado nos últimos anos, e os resultados apresentam algumas inconsistências. Há estudos que apontam que as vizinhanças com menor nível socioeconômico têm associação com o sono de curta duração ^7^ ^,^ ^8^ ^,^ ^9^. Além disso, existem evidências de associação entre vizinhanças com alto nível de desemprego e maior frequência de insônia ^10^. Por outro lado, os resultados de alguns estudos sugerem que não há associação do nível socioeconômico da vizinhança com a duração do sono ^11^ ^,^ ^12^ e a insônia ^6^.

Estudos que examinaram a relação entre as características da vizinhança e o sono mostraram que as condições ambientais (sons, luzes, temperatura, umidade e atmosfera), o ambiente social (coesão social, segurança, violência e desordem) e as características do ambiente físico da vizinhança (área para caminhada, espaço verde e densidade populacional) podem afetá-lo ^13^ ^,^ ^14^. Há fortes evidências para a associação de ruído ambiental, falta de coesão social e sensação de insegurança com problemas de sono ^15^.

A desvantagem social da vizinhança, expressa pela falta de recursos econômicos e sociais, captura os aspectos dos ambientes de moradia e incluem dimensões adicionais de marginalização social ^16^. A desigualdade social caracterizada pela diferença entre as vizinhanças é conhecida como segregação residencial, que se refere à separação física dos indivíduos pela distribuição espacial da população na área metropolitana de acordo com a raça e/ou condição socioeconômica ^17^ ^,^ ^18^.

A segregação residencial socioeconômica, em particular de renda, implica que as famílias mais pobres residem em vizinhanças com renda média mais baixa do que as famílias mais ricas ^19^. Evidências demonstraram que a segregação residencial está associada a desfechos negativos de saúde ^20^ ^,^ ^21^. No Brasil, Barber et al. ^21^ identificaram que indivíduos que moravam em vizinhanças economicamente segregadas eram mais propensos a terem hipertensão e diabetes mellitus.

Apesar do conhecimento sobre alguns aspectos da vizinhança modificarem o sono, até esse momento a relação entre a segregação residencial socioeconômica e o sono não foi explorada na literatura. Portanto, o objetivo deste estudo foi verificar essa associação (duração e privação do sono, insônia, sonolência diurna e dois ou mais problemas do sono). Nossa hipótese é que indivíduos que moram em vizinhanças com alta segregação residencial socioeconômica têm mais chance de disporem de sono de curta duração, queixas de insônia, privação do sono e sonolência diurna, bem como mais chance de apresentarem dois ou mais problemas do sono concomitantemente.

## Métodos

### População do estudo

O *Estudo Longitudinal de Saúde do Adulto* (ELSA-Brasil) é um estudo de coorte, composto por servidores públicos ativos e aposentados de ambos os sexos e com idades entre 35 e 74 anos, pertencentes a sete instituições públicas (Universidade Federal da Bahia - UFBA, Universidade Federal do Espírito Santo - UFES, Universidade Federal de Minas Gerais - UFMG, Universidade Federal do Rio Grande do Sul - UFRGS, Universidade de São Paulo - USP, Centro Federal de Educação Tecnológica de Minas Gerais - CEFET-MG, e Fundação Oswaldo Cruz - FIOCRUZ) ^22^.

A população da linha de base foi constituída de 15.105 servidores públicos que se voluntariaram ou foram ativamente recrutados, com o objetivo de preencher cotas específicas de sexo, idade e categoria ocupacional para garantir variabilidade amostral ^22^. O estudo foi aprovado pelos Comitês de Pesquisa e Ética das instituições envolvidas (Instituto de Saúde Coletiva/UFBA - 0017.1.069.000-06; FIOCRUZ - 0058.0.011.000-07; Hospital Universitário/USP - 0016.1.198.000-06; UFMG - 0186.1.203.000-06; Centro de Ciências da Saúde/UFES - 08109612.7.2003.5060; e Hospital de Clínicas de Porto Alegre/UFRGS - 48608515.5.1001.5327) e todos os participantes assinaram o termo de consentimento livre e esclarecido.

Para este estudo, foram utilizados os dados da segunda visita de entrevistas e exames (Onda 2), que ocorreu entre 2012 e 2014. Nesse período, foram reavaliados 14.014 participantes (6.357 homens e 7.657 mulheres) e o questionário multidimensional foi aplicado com o intuito de atualizar os dados obtidos na linha de base; na Onda 2, foram inseridas questões sobre o sono ^23^ ^,^ ^24^. O questionário foi aplicado em entrevista face-a-face realizada por equipe treinada e certificada, seguindo rigoroso padrão de garantia e controle de qualidade ^23^ ^,^ ^24^.

Foram excluídos das análises os participantes com dados faltantes nas variáveis demográficas, socioeconômicas (1,6%) e relacionadas ao sono (1,1%). Para a variável de segregação residencial socioeconômica, também foram excluídos os que não moravam nas capitais onde ficavam localizados os centros de pesquisa (22,7%). Além disso, foram excluídos quem relatou duração do sono de < 3 horas ou > 12 horas (0,4%) ^25^, assim como aqueles que se autodeclararam com raça/cor amarela ou indígenas, devido ao pequeno número de participantes em cada categoria (2,5% e 1%, respectivamente). Após a exclusão de 4.096 participantes (29,2%), a amostra final foi composta por 9.918 pessoas.

### Variáveis selecionadas

#### Variável de exposição: segregação residencial socioeconômica

O registro dos endereços permitiu o georreferenciamento e a consideração dos dados de vizinhança no estudo. O recorte geográfico, aqui chamado de vizinhança, foi baseado em limites definidos pelo estudo e criados para cada local usando um método de agregação espacial baseado no Spatial ‘K’luster Analysis by Tree Edge Removal (SKATER). Esse método foi utilizado para criar aglomerados de setores censitários contíguos com população mínima de 5 mil habitantes e homogêneos em relação a quatro indicadores socioeconômicos do *Censo Demográfico* de 2010 do Instituto Brasileiro de Geografia e Estatística (IBGE) ^26^: proporção da população 0-4 anos de idade; tamanho do agregado familiar; média da renda familiar; e a proporção de brancos. As vizinhanças foram definidas nas localidades dentro das fronteiras metropolitanas dos seis centros de investigação ELSA-Brasil ^27^.

Para o cálculo da medida de segregação residencial socioeconômica, foi utilizada a estatística Getis-Ord Local Gi* (estatística Gi*) ^28^, uma abordagem espacial que tem sido usada em investigações epidemiológicas de segregação residencial e doença cardiovascular ^20^. A estatística Gi* é um escore z ponderado espacialmente que representa o quanto uma composição de renda da vizinhança se desvia da média área metropolitana.

Além disso, essa estatística considera o agrupamento espacial da segregação (ou seja, vizinhanças pobres cercadas por outras vizinhanças pobres terão valores maiores) e a composição da renda da área metropolitana em que a vizinhança está inserida. Isso é particularmente importante porque uma vizinhança com um valor de composição de 50% de pobres significa algo muito diferente em termos de segregação: se 5% do total da população são pobres, a vizinhança é altamente segregada; entretanto, se 50% do total da população são pobres, a vizinhança não é segregada ^21^.

Neste estudo, a estatística Gi* foi baseada na proporção de chefes de família em uma vizinhança que ganha uma renda mensal de 0-3 salários mínimos (ou seja, até três salários mínimos ou aproximadamente US$ 900 em 2010) e derivada dos dados do *Censo Demográfico* de 2010 do IBGE ^26^. A decisão de usar três salários mínimos como ponto de corte foi baseada em estudos anteriores de segregação socioeconômica no Brasil ^29^.

Altas pontuações positivas representam vizinhanças que são mais segregadas (ou seja, maior proporção de domicílios com 0-3 salários mínimos), enquanto escores negativos representam vizinhanças menos segregadas (ou seja, menor proporção de domicílios com 0-3 salários mínimos). Com base na distribuição da amostra, as categorias de segregação que representaram as vizinhanças com escores de segregação ± 1 desvio padrão (DP) acima da média metropolitana são: alta (estatística Gi* > 1), média (estatística Gi* entre 1 e -1) e baixa (estatística Gi* < -1) ^21^.

#### Variáveis de desfecho

(1) Duração do sono - mensurada por meio da pergunta “Quantas horas, em média, o(a) senhor(a) dorme numa noite habitual de sono?”. Os participantes foram classificados em três grupos: duração curta do sono (≤ 6 horas), duração adequada do sono (> 6 e < 9 horas) e longa duração do sono (≥ 9 horas) ^11^. A pergunta apresentou uma boa confiabilidade teste-reteste, avaliada em uma subamostra (coeficiente de correlação intraclasse - CCI: 0,761; intervalo de 95% de confiança - IC95%: 0,685; 0,819) ^24^.

(2) Queixas de insônia - avaliados pelas perguntas a seguir que foram precedidas pela frase “Agora, gostaríamos de saber com que frequência (nunca, raramente, às vezes, quase sempre, sempre) nas últimas trinta noites, o(a) senhor(a)”: “teve dificuldade em pegar no sono?”, “acordou durante o sono e teve dificuldade para dormir de novo?” e “acordou antes da hora desejada e não conseguiu adormecer novamente?”. Os participantes que relataram “quase sempre” ou “sempre” em uma das três queixas descritas acima foram classificados como indivíduos com insônia ^30^. Valores de kappa = 0,759 (IC95%: 0,651; 0,867) foram identificados na confiabilidade teste-reteste para as queixas de insônia ^24^.

(3) Privação do sono - baseada na seguinte pergunta: “Quantas horas o(a) senhor(a) gostaria de dormir para se sentir recuperado(a)?”. Foi calculada a diferença do número de horas que o indivíduo relatou que habitualmente dorme e o número de horas que relatou precisar dormir para se sentir recuperado. Os participantes foram classificados em dois grupos: sem privação do sono (aqueles que dormem o suficiente para se sentirem recuperados) ou com privação do sono (aqueles com a diferença maior que uma hora em relação ao número de horas que dorme e o número de horas que precisaria dormir para se sentir recuperado) ^31^.

(4) Sonolência diurna: avaliada pela pergunta: “O(A) senhor(a) se sente frequentemente cansado(a), fatigado(a) ou sonolento(a) durante o dia?”. Foram classificados com sonolência diurna os que responderam sim e sem aqueles que responderam não.

(5) Dois ou mais problemas associados do sono: variável obtida a partir do número referido de problemas de sono (curta duração, sonolência diurna, insônia e privação de sono), classificando em “sem problemas do sono” aqueles que relataram até um e “com problemas de sono” para aqueles que relataram dois ou mais.

#### Covariáveis: variáveis demográficas e socioeconômicas

Foram utilizadas as seguintes variáveis: sexo, idade (anos), raça/cor autodeclarada de acordo com as categorias do *Censo Demográfico* (preta, parda, branca) e situação conjugal (unido [casado e vive em união] e não unido [separado, divorciado, viúvo e solteiro]). A escolaridade foi categorizada em baixa (Ensino Médio incompleto ou menos), média (Ensino Médio completo), alta (Ensino Superior completo com ou sem Pós-graduação *lato sensu*) e muito alta (Mestrado e Doutorado). Renda familiar *per capita* (calculada como a renda familiar total dividida pelo número de dependentes da renda) foi categorizada em 1 a 3 salários mínimos ou > 3 salários mínimos.

### Análise estatística

Foi realizada análise descritiva das variáveis. Para as contínuas, os valores foram expressos pela média e DP; e para variáveis categóricas, em frequências absoluta e relativa.

Para examinar as estimativas de *odds ratio* (OR) e o IC95% da segregação residencial socioeconômica baixa/média/alta associada às categorias de duração do sono (curta *vs.* normal, longa *vs.* normal), foram aplicados modelos de regressão logística multinomial. Nos desfechos de queixas de insônia, privação do sono, sonolência diurna e dois ou mais problemas de sono, foram utilizados modelos de regressão logística binomial.

No primeiro modelo, ajustamos para idade e sexo. No segundo, ajustamos adicionalmente para raça/cor autodeclarada, situação conjugal, escolaridade e renda familiar *per capita*. Para verificar a multicolinearidade, foram examinados os fatores de inflação de variância (VIF) em cada modelo (variáveis: sexo, idade, raca/cor, situação conjugal, escolaridade, renda familiar *per capita* e segregação residencial socioeconômica), e nenhuma multicolinearidade relativa (VIF < 10) foi observada. Os dados foram analisados utilizando o software R versão 3.5.1 (http://www.r-project.org).

## Resultados

A população deste estudo foi composta por 9.918 participantes (56,1% de mulheres), com média de idade de 56 anos. A maioria se autodeclarou branco (56,7%), casado (63,6%) e com escolaridade alta (31,9%) ou muito alta (31,3%), além de renda familiar *per capita* > 3 salários mínimos (63,2%) (Tabela 1).

**Tabela 1:** Prevalência dos fatores demográficos e socioeconômicos. *Estudo Longitudinal de Saúde do Adulto* (ELSA-Brasil), 2012-2014.

Variável	n	%
Sexo		
Masculino	4.347	43,8
Feminino	5.571	56,2
Idade em anos (média ± DP)	56 ± 9		
Raça/Cor		
Preta	1.545	15,6
Parda	2.750	27,7
Branca	5.623	56,7
Situação conjugal		
Casado	6.306	63,6
Solteiro	3.612	36,4
Escolaridade		
Baixa	850	8,6
Média	2.799	28,2
Alta	3.167	31,9
Muito alta	3.102	31,3
Renda *per capita* (salários mínimos)		
≤ 3	3.654	36,8
> 3	6.264	63,2
Segregação residencial socioeconômica		
Baixa	5.839	58,9
Média	2.691	27,1
Alta	1.388	14,0

DP: desvio padrão.

A média da duração do sono foi de 6,5 ± 1,3 horas. Quase metade dos participantes foram classificados com sono de curta duração (49,6%), com apenas 3,4% classificados com sono de longa duração (Tabela 2). Além disso, 23,2% relataram queixas de insônia, 45,7% privação do sono, 42,5% sonolência diurna e 48,6% foram classificadas com ≥ 2 problemas de sono (Tabela 3). A prevalência de sono de curta duração foi maior entre os homens. Por outro lado, as mulheres apresentaram maior prevalência de insônia, privação de sono, sonolência diurna e ≥ 2 problemas de sono. Notavelmente e de forma consistente, os pretos, seguidos pelos pardos, solteiros, com menor escolaridade e menor renda tiveram maior prevalência de todos os problemas de sono (Tabelas 2 e 3).

**Tabela 2:** Prevalência da duração do sono de acordo com os fatores demográficos, socioeconômicos e classificação da segregação residencial socioeconômica. *Estudo Longitudinal de Saúde do Adulto* (ELSA-Brasil), 2012-2014.

Variável	Duração do sono (horas)					
	≤ 6		6-9		≥ 9	
	n	%	n	%	n	%
Sexo						
Masculino	2.106	48,4	2.135	49,1	106	2,4
Feminino	2.557	45,9	2.786	50,0	228	4,1
Raça/Cor						
Preta	905	58,6	575	37,2	65	4,2
Parda	1.414	51,4	1.242	45,2	94	3,4
Branca	2.344	41,7	3.104	55,2	175	3,1
Situação conjugal						
Casado	2.878	45,6	3.224	51,1	204	3,3
Solteiro	1.785	49,4	1.697	47,0	130	3,6
Escolaridade						
Baixa	438	51,5	361	42,5	51	6,0
Média	1.423	50,8	1.247	44,6	129	4,6
Alta	1.489	47,0	1.587	50,1	91	2,9
Muito alta	1.313	42,3	1.726	55,7	63	2,0
Renda *per capita* (salários mínimos)						
≤ 3	1.924	52,6	1.578	43,2	152	4,2
> 3	2.739	43,7	3.343	53,4	182	2,9
Segregação residencial socioeconômica						
Baixa	2.559	43,8	3.105	53,2	175	3,0
Média	1.359	50,5	1.241	46,1	91	3,4
Alta	745	53,7	575	41,4	68	4,9
Total	4.663	49,6	4.921	47,0	334	3,4

**Tabela 3:** Prevalência dos desfechos do sono de acordo com os fatores demográficos, socioeconômicos e classificação da segregação residencial socioeconômica. *Estudo Longitudinal de Saúde do Adulto* (ELSA-Brasil), 2012-2014.

Variável	Queixas de insônia		Privação do sono		Sonolência diruna		≥ 2 problemas associados ao sono	
	n	%	n	%	n	%	n	%
Sexo								
Masculino	823	18,9	1.715	39,4	1.548	35,6	1.888	43,4
Feminino	1.474	26,4	2.816	50,5	2.667	47,8	2.937	52,7
Raça/Cor								
Preta	417	27,0	872	56,4	719	46,5	909	58,8
Parda	702	25,5	1.388	50,5	1.238	45,0	1.453	52,8
Branca	1.178	20,9	2.271	40,4	2.258	40,1	2.463	43,8
Situação conjugal								
Casado	1.329	21,1	2.790	44,4	2.565	40,6	2.949	46,7
Solteiro	968	26,8	1.741	48,2	1.650	45,6	1.876	51,9
Escolaridade								
Baixa	274	32,2	393	46,2	338	39,7	446	52,5
Média	734	26,2	1.497	53,5	1.293	46,2	1.544	55,1
Alta	701	22,1	1.513	47,8	1.408	44,4	1.590	50,2
Muito alta	588	18,9	1.128	36,4	1.176	37,9	1.245	40,1
Renda *per capita* (salários mínimos)								
≤ 3	971	26,6	1.948	41,2	1.716	46,9	2.055	56,2
> 3	1.326	21,2	2.583	53,3	2.499	39,9	2.770	44,2
Segregação residencial socioeconômica								
Baixa	1.246	21,3	2.397	41,1	2.375	40,6	2.598	44,5
Média	681	25,3	1.385	51,5	1.169	43,4	1.430	53,1
Alta	370	26,6	749	53,9	671	48,3	797	57,4
Total	2297	23,2	4.531	45,7	4.215	42,5	4.825	48,6

Em relação a distribuição da segregação residencial, 58,9% residiam em vizinhanças de baixa segregação, 27,1% em regiões de média e 14% de alta segregação (Tabela 1). Entre os indivíduos que moravam em áreas de alta segregação residencial socioeconômica, 26% eram pretos, 19% pardos, 8% brancos e a maioria tinha baixa escolaridade e renda (Tabela 4).

**Tabela 4:** Prevalência da segregação residencial socioeconômica, de acordo com os fatores demográficos, socioeconômicos e desfechos do sono. *Estudo Longitudinal de Saúde do Adulto* (ELSA- Brasil), 2012-2014.

Variável	Duração do sono (horas)					
	≤ 6		6-9		≥ 9	
	n	%	n	%	n	%
Sexo						
Masculino	2.502	57,6	1.200	27,6	645	14,8
Feminino	3.337	59,9	1.491	26,8	743	13,3
Idade em anos (média ± DP)	56 ± 9			54 ± 8		55 ± 8	
Raça/Cor						
Preta	557	36,1	586	37,9	402	26,0
Parda	1.317	47,9	900	32,7	533	19,4
Branca	3.965	70,5	1.205	21,4	453	8,1
Situação conjugal						
Casado	3.650	57,9	1.751	27,8	905	14,3
Solteiro	2.189	60,6	940	26,0	483	13,4
Escolaridade						
Baixa	199	23,4	364	42,8	287	33,8
Média	1.009	36,1	1.103	39,4	687	24,5
Alta	1.965	62,0	873	27,5	329	10,4
Muito alta	2.666	85,9	351	11,3	85	2,8
Renda *per capita* (salários mínimos)						
≤ 3	1.262	34,5	1.415	38,7	977	26,8
> 3	4.577	73,1	1.276	20,4	411	6,5
Duração do sono						
Curto	3.559	54,9	1.359	29,1	745	16,0
Normal	3.105	63,1	1.241	25,2	575	11,7
Longo	175	52,4	91	27,2	68	20,4
Queixas de insônia						
Não	4.593	60,3	2.010	26,4	1.018	13,3
Sim	1.246	54,2	681	29,7	370	16,1
Privação do sono						
Não	3.442	63,9	1.306	24,2	639	11,9
Sim	2.397	52,9	1.385	30,6	749	16,5
Sonolência diurna						
Não	3.464	60,7	1.522	26,7	717	12,6
Sim	2.375	56,4	1.169	27,7	671	15,9
≥ 2 problemas associados ao sono						
Não	3.241	63,6	1.261	24,8	591	11,6
Sim	2.598	53,9	1.430	29,6	797	16,5

DP: desvio padrão.

Os indivíduos que vivem em vizinhanças de alta segregação residencial socioeconômica dormiram em média 14 minutos a menos que aqueles que viviam em vizinhanças de baixa segregação residencial socioeconômica. Além disso, participantes que residiam em áreas de alta segregação residencial socioeconômica apresentaram maior prevalência de sono de curta e longa duração (53,6% e 4,9% respectivamente), queixas de insônia (26,6%), privação do sono (53,9%), sonolência diurna (48,3%) e ≥ 2 problemas associados do sono (57,4%). Foi possível observar um claro padrão de gradiente, em que todos os desfechos de sono aumentaram conforme o aumento da segregação residencial socioeconômica (Tabela 4). A associação entre essa alta e duração curta do sono foi atenuada, porém se manteve significativa após o ajuste pelas variáveis socioeconômicas (OR = 1,22; IC95%: 1,07; 1,40). A duração longa do sono teve associação com a alta segregação residencial socioeconômica apenas no modelo 1, perdendo a significância estatística após a inclusão das variáveis de ajuste do modelo 2 (OR = 1,33; IC95%: 0,95; 1,86). O mesmo padrão foi observado para as queixas de insônia (OR = 1,08; IC95%: 0,93; 1,26) (Tabela 5).

**Tabela 5:** Associações entre a segregação residencial socioeconômica e os desfechos de sono. *Estudo Longitudinal de Saúde do Adulto* (ELSA-Brasil), 2012-2014.

Segregação residencial socioeconômica	Duração do sono		Queixas de insônia	Privação do sono	Sonolência diurna	≥ 2 problemas associados do sono
	Curta	Longa				
	OR (IC95%)	OR (IC95%)	OR (IC95%)	OR (IC95%)	OR (IC95%)	OR (IC95%)
Modelo 1						
Baixa	1,00	1,00	1,00	1,00	1,00	1,00
Média	1,32 (1,20; 1,45)	1,38 (1,06; 1,80)	1,29 (1,15; 1,43)	1,45 (1,32; 1,59)	1,08 (0,99; 1,19)	1,37 (1,25; 1,51)
Alta	1,56 (1,39; 1,77)	2,24 (1,67; 3,01)	1,39 (1,21; 1,59)	1,64 (1,45; 1,85)	1,35 (1,20; 1,52)	1,66 (1,47; 1,87)
Modelo 2						
Baixa	1,00	1,00	1,00	1,00	1,00	1,00
Média	1,13 (1,02; 1,25)	0,95 (0,71; 1,26)	1,09 (0,96; 1,22)	1,17 (1,05; 1,29)	0,99 (0,89; 1,09)	1,12 (1,02; 1,24)
Alta	1,22 (1,07; 1,40)	1,33 (0,95; 1,86)	1,08 (0,93; 1,26)	1,20 (1,05; 1,37)	1,17 (1,03; 1,34)	1,24 (1,08; 1,41)

IC95%: intervalo de 95% de confiança; OR: *odds ratio*.

Nota: Modelo 1 - ajustado por sexo e idade; Modelo 2 - ajustado por sexo, idade, raça/cor, situação conjugal, escolaridade e renda familiar *per capita*.

Para os demais desfechos de sono, as associações se mantiveram significativas após ajustes. Assim, os participantes que viviam em vizinhanças de alta segregação residencial socioeconômica tiveram maior chance de ter privação do sono (OR = 1,20; IC95%: 1,05; 1,37), sonolência diurna (OR = 1,17; IC95%: 1,03; 1,34) e ≥ 2 problemas associados do sono (OR = 1,24; IC95%: 1,08; 1,41) quando comparados com os participantes que viviam em vizinhanças de baixa segregação. Vale destacar que houve um aumento gradual da OR de desfechos negativos de sono da média segregação residencial socioeconômica para alta, exceto para queixas de insônia no modelo ajustado (Tabela 5).

## Discussão

Este foi o primeiro estudo que investigou a associação entre segregação residencial socioeconômica e múltiplas dimensões do sono no Brasil. Nossa hipótese foi parcialmente confirmada: a alta segregação residencial socioeconômica foi associada à duração curta e longa do sono, à queixas de insônia, à privação do sono, à sonolência diurna e à ≥ 2 problemas do sono após ajuste para sexo e idade. Porém, ao ajustar para outros fatores sociodemográficos, a associação não foi significativa para a duração longa do sono e para as queixas de insônia. Em geral, com o aumento da segregação residencial socioeconômica, também houve aumento da prevalência dos desfechos negativos do sono.

Em relação a duração do sono, indivíduos que moram em vizinhanças com média e alta segregação residencial socioeconômica tiveram, respectivamente, 13% e 22%, mais chance de terem um sono de curta duração (≤ 6 horas) do que aqueles que moram em áreas de baixa segregação residencial socioeconômica.

Não foram encontradas pesquisas sobre a segregação residencial socioeconômica e o sono para comparação dos resultados. Entretanto, estudos prévios verificaram a associação entre o nível socioeconômico da vizinhança e o sono de curta duração nos Estados Unidos. O nível socioeconômico mais baixo da vizinhança foi associado ao maior risco relativo de relatar sono muito curto (< 5 horas) e curto (5-6 horas) em um estudo longitudinal ^9^. Já nos transversais, foi demonstrado que viver em vizinhanças de baixo nível socioeconômico foi associado a maior chance de sono muito curto (< 5 horas) e curto (5-6,9 horas) ^7^ e também a menor duração do sono ^8^. Vale ressaltar que o indicador de segregação residencial socioeconômica mensura a diferença entre as vizinhanças da mesma área urbana, todavia, as investigações supracitadas utilizaram apenas indicadores de nível socioeconômico delas.

Alguns fatores potenciais podem vincular os aspectos da vizinhança com o sono. Primeiro, o ambiente social dessa pode influenciar o sono indiretamente por meio da ativação dos sistemas de estresse do corpo ^6^. Por exemplo, o medo de crime e violência pode aumentar a ansiedade ou outros aspectos psicológicos, tendo potencial de levar à desregulação do sistema nervoso simpático, levar a hiperexcitação e vigilância e reduzir o tempo disponível para dormir ^6^ ^,^ ^13^.

O ambiente físico, englobador da arquitetura e do planejamento da cidade como a conectividade das ruas, calçadas, áreas residenciais e comerciais, disponibilidade de alimentos saudáveis e espaços sociais (p.ex.: igrejas), pode afetar a mobilidade urbana, a atividade física, a coesão social e, consequentemente, o sono ^14^. Similarmente, outros fatores ambientais físicos (ruído e tráfego nas ruas) podem prejudicar essa atividade de forma mais direta, perturbando o repouso devido aos distúrbios nas proximidades ^6^. Viver nessas áreas pode desencadear a liberação de hormônios do estresse como cortisol e epinefrina, que promovem a excitação mental e fisiológica em detrimento do sono ^8^.

A exposição às condições ambientais adversas tende a ser padronizada por raça e *status* socioeconômico, o que perpetua as disparidades de saúde e, particularmente, as da saúde do sono ^13^. A segregação residencial é a representação espacial das desigualdades raciais e socioeconômicas, consequência das estruturas de governança, trabalho e iniquidades, marcantes no Brasil ^21^ ^,^ ^32^. A separação espacial com base na classe social e na raça não se trata apenas da segregação das pessoas, mas também dos recursos econômicos e sociais necessários para alcançar a saúde ideal ^21^.

Além disso, a exposição sistemática e desproporcional de pretos e pardos a ambientes de bairros socioeconomicamente segregados reflete a longa história de desigualdades raciais no Brasil, e pode ser uma forma de racismo estrutural que perpetua essas desigualdades em saúde no país ^21^. Recentemente, Guimarães et al. ^33^ demonstraram que viver em vizinhanças com maior segregação socioeconômica foi associado à pior percepção de saúde em todos os grupos raciais, sendo ainda mais fortes entre pretos e pardos.

Por outro lado, não houve correlação entre as queixas de insônia com a alta segregação residencial socioeconômica após ajustes, divergindo do estudo de Riedel et al. ^10^ em que a chance de relatar insônia aumentou nos indivíduos que viviam em vizinhanças com alto índice de desemprego.

Outras pesquisas não encontraram relação entre a duração curta ^6^ ^,^ ^11^ ^,^ ^12^ e longa do sono ^11^ ^,^ ^12^, a sonolência diurna ^11^ e a insônia ^6^ com o baixo nível socioeconômico da vizinhança. Vale ressaltar que esses tiveram menor tamanho de amostra quando comparados com os que evidenciaram a associação entre os desfechos do sono e o nível socioeconômico da vizinhança.

Até onde se pode investigar, esse foi a primeira análise que demonstrou a conexão da alta segregação residencial socioeconômica da vizinhança com a privação do sono, a sonolência diurna e ≥ 2 problemas associados ao sono. Como pontos fortes deste estudo, é possível apontar o grande tamanho da amostra com ampla diversidade sociodemográfica, relevante variação geográfica (seis capitais estaduais das regiões Nordeste, Sudeste e Sul do Brasil), ambientes urbanos com diversidade socioeconômica das vizinhanças, e medidas quantitativas e qualitativas do sono.

Contudo, podemos citar as limitações apresentadas, incluindo o uso da duração do sono autorreferida, que está sujeita a erro de medição. No entanto, essa é amplamente utilizada em estudos epidemiológicos em muitos países. Em nossa população amostral, esse desfecho apresentou boa confiabilidade teste-reteste em uma subamostra, apoiando a validação de nossos achados. Outro fato é que o modelo 2 poderia ser considerado “sobre ajustado” por variáveis sociodemográficas (renda e escolaridade), porém elas foram mantidas por serem individuais e porque a segregação residencial socioeconômica representa uma medida de nível da vizinhança.

Além disso, o ELSA-Brasil envolve apenas funcionários públicos, não incluindo indivíduos nos extremos da distribuição de renda brasileira. Dessa forma, a amostra não é totalmente representativa da população brasileira e os nossos resultados podem não ser generalizáveis para áreas não urbanas. Por fim, neste estudo foram utilizados apenas dados da 2ª onda, portanto, não é possível confirmar a existência de uma relação causal entre a segregação residencial socioeconômica e os desfechos do sono.

Com base nas informações anteriores, uma melhor compreensão da segregação residencial socioeconômica no Brasil poderia potencialmente informar políticas e/ou programas, refletindo a distribuição desigual de privações e privilégios, especialmente em um momento em que o progresso na redução de disparidades está ameaçado ^32^.

## Conclusão

Viver em vizinhanças com alta segregação residencial socioeconômica aumenta a chance de se ter duração curta do sono, privação do sono, sonolência diurna e ≥ 2 problemas associados ao sono. Com os resultados deste estudo, foi possível verificar que as dimensões da saúde do sono não estão distribuídas de forma equitativa pela população brasileira e que isso reforça a associação negativa entre elas e segregação residencial socioeconômica. Políticas públicas de saúde poderiam prover recursos para melhorar a saúde do sono nas populações mais vulneráveis, melhorar as condições ambientais de moradia, além de reduzirem as desigualdades socioeconômicas que são tão evidentes no Brasil.
